# Effects of stress conditions on plasma parameters and gene expression in the skin mucus of farmed rainbow trout (*Oncorhynchus mykiss*)

**DOI:** 10.3389/fvets.2023.1183246

**Published:** 2023-09-06

**Authors:** Elisa Fiordelmondo, Gian Enrico Magi, Adina Friedl, Mansour El-Matbouli, Alessandra Roncarati, Mona Saleh

**Affiliations:** ^1^School of Bioscience and Veterinary Medicine, University of Camerino, Camerino, Italy; ^2^Clinical Division of Fish Medicine, University of Veterinary Medicine, Vienna, Austria

**Keywords:** fish welfare, fish farming, qRT-PCR, cortisol, IL-6

## Abstract

The aim of this study was to investigate the physiological response of rainbow trout (*Oncorhynchus mykiss*) before slaughtering in the last phase of farming analyzing skin mucus and plasma. Two groups of rainbow trout were considered: Group UN (“unstressed”), represented by fish randomly captured from raceways, in the last phase of a standard fattening cycle; Group S (“stressed”), collected at the end of the pre-slaughtering tank, soon after slaughtering. The fish skin mucus was swabbed from head to tail using a sterile plastic spatula and the blood was collected through an endocardial puncture. qRT-PCR was used to study the gene expression in skin mucus. The mRNA expression levels of the IL-6 and IgD genes were higher in the S than in the Group UN. The plasma analysis showed an only a decrease in the glucose plasma levels in the Group S when compared to the Group UN. The present results indicated that the procedures adopted after slaughtering only affected changes in plasma glucose and skin mucus activity in rainbow trout suggesting that management protocol was compatible with non-stressful farming conditions.

## 1. Introduction

In aquaculture, handling, food deprivation, and very high stocking density can induce stress (1). The alteration of health status of farmed fish can be evaluated by analyzing plasma parameters (2–6). Stress conditions could modify the production of skin mucus and its composition, negatively affecting fish welfare (7). Stress biomarkers can be present in skin mucus which is considered a non-invasive stress indicator in fish; skin mucus is a key natural barrier that protects fish from potential microbiological attacks caused by pathogens originating in the environment in which they live (8–10) The skin mucus plays an essential role in preventing parasitism by bacteria and fungi, acting as a chemical defense barrier in fish (11–13). Secreted mucus contains elements of the immune system (specific immunoglobulins) and can change in response to external stimuli and stress (14–17).

In farming, rainbow trout (*Oncorhynchus mykiss*) are most exposed to stressful conditions when fish are moved to smaller tanks (18) with higher density than the breeding tanks. After the required fasting time (19), fish are caught using a pumping system and undergo stunning before being finally slaughtered.

This study aimed to assess the welfare of farmed rainbow trout at the end of the farming cycle. To evaluate potential differences with fish reared in the raceways, the expression of several immunity genes, involved in stress response and related to T and B cells, and the main plasma parameters were examined.

## 2. Materials and methods

### 2.1. Farming conditions and sampling

Rainbow trout were sampled from the fattening farm of a company located in the Italian Central Apennine area, based on 1,000-L in parallel raceways (120 m^3^ each) containing circulating groundwater. The inlet water came to the farm system with a constant velocity of 0.25 m/s and flowed through four parallel raceways. Water quality was daily checked for dissolved oxygen (8.5 ± 1.5 mg/L), temperature (10–12°C), pH (7.8 ± 0.4), ammonia (0.16 ± 0.2 mg/L), nitrites (0.05 ± 0.01 mg/L), and nitrates (1.15 ± 0.6 mg/L). Fish were fed with extruded feed (prot. 44%; lip. 22% as it is), produced by the same trout farm company, distributed with a semi-moving wagon up to levels close to satiety at around the same time every day, twice a day. For the trial, were considered: Group UN, representing the control group, composed by 10 fish randomly reared from the last portion of raceways, using the same net and the same capture procedure on a daily basis; Group S represented by 10 fish, collected at the end of the production line, soon after slaughtering. The fish were subsequently placed in a basin containing water from the same raceways and anesthetic, essential oils extracted from cloves (C8392, Merck, Germany), at the concentration of 0.04 mg/L. The stress-causing agents represented usual procedures encountered in a rainbow trout farm, including fish capture and transfer from the raceways (20 kg/m^3^) to the pre-slaughtering tank. The trout remain in a fasted state for 4–6 h in high density (35 kg/m^3^). Fish were transferred by means of pumping pipe to the stunning tanks (20 kg/m^3^) where fish lose consciousness before being slaughtered. Groups UN and S had about the same commercial size (around 350 g).

### 2.2. Skin mucus sampling

Body surface was individually swabbed from head to tail using a sterile plastic spatula from the anterodorsal to the posterior surface, without including the ventral portion. Prior to the skin mucus sampling, general health status of each fish was examined. Then, the pure mucus was sampled in clean Eppendorf® tubes, immediately frozen in dry ice, and sent to the Department of Fish Clinic of Vienna University in dry ice bags.

### 2.3. Blood sampling

Blood sampling was performed by endocardial puncture, using a new syringe for each fish. After having, removed the needle from the syringe, the blood samples (about 1–2 cc) were placed in tubes containing a drop of anticoagulant (heparin) and sent to UNICAM laboratories. The blood samples underwent centrifugation for 20 min at 3,000 RPM (SI-TRON TINCA 3003 M). The obtained supernatant represented the plasma (0.5–1 cc), which was collected with a pipette and placed in individual tubes for subsequent analyses using spectrophotometric equipment (BT3500VET, Microtech 648 Electrophoresis) to determine the parameters of interest. For the present study, plasma parameters were quantified according to international methods as described below. Glucose was determined by enzymatic colorimetric GOD-PAP test based on glucose oxidase peroxidase reaction at 546 nm (linearity up to 400 mg/dL), using commercial kit (ref. 244 L, Biotecnica Instruments, Rome, Italy). Total proteins were determined by photometric test according to Biuret method at 546 nm (linearity up to 15 g/dL; kit ref. 304 L, Biotecnica Instruments, Rome, Italy). Cholesterol was determined by colorimetric enzymatic CHOD-PAP test at 546 nm (linearity up to 750 mg/dL; kit ref. 135 L, Biotecnica Instruments, Rome, Italy). Triglycerides were essayed by colorimetric enzymatic GPO-PAP test at 546 nm (kit ref. 315 L, Biotecnica Instruments, Rome, Italy). Albumins were essayed by colorimetric endpoint increasing reaction BCG test at 600 nm (linearity up to 6 g/dL, kit ref. 424 L, Biotecnica Instruments, Rome, Italy). Globulins were determined by difference between total proteins and albumins. The plasma cortisol level was determined using commercial enzyme immunoassay (ELISA) kit (cortisol ELISA RE52061 IBL International GmbH, Hamburg, Germany).

### 2.4. RNA extraction and qRT-PCR analysis

In the Department of Fish Clinic, RNA extraction from all skin mucus samples was performed following the RNeasy tissue kit manual of instructions (Qiagen, Hilden, Germany). In short, samples were lysed in RLT buffer containing β-mercaptoethanol. Steel beads were then added to the sample and homogenized using TissueLyser II (Qiagen) for 3 min at 25 Hz. Finally, RNA concentration was quantified using a NanoDrop 1000 spectrophotometer (LabTech International). Reverse transcription was performed using iScript cDNA Synthesis Kit (Bio-Rad, Hercules, CA, United States) on 1 μg of total RNA isolated from Group UN and Group S samples. Finally, RNA were eluted from the columns in RNase-free water and stored at −80°C before use. PCR primers specific to the target genes were selected based on the scientific literature, and their nucleotide sequences are displayed in Table 1.

**Table 1:** List of qRT-PCR primers used in the present study.

Nr.	Oligoname	Forward sequence (5′- > 3′)	Reverse sequence	Reference
1	CD8	AGTCGTGCAAAGTGGGAAAG	GGTTGCAATGGCATACAGTG	(20)
2	IL-6	TTTCAGAAGCCCGTGGAAGAGA	TCTTTGACCAGCCCTATCAGCA	(21)
3	IL-10a	GGATTCTACACCACTTGAAGAGCC	GTCGTTGTTGTTCTGTGTTCTGTTGT	(22)
4	IL-8	AGAGACACTGAGATCATTGCCAC	CCCTCTTCATTTGTTGTTGGC	(21)
5	CD4	CCTGCTCATCCACAGCCTAT	CTTCTCCTGGCTGTCTGACC	(20)
6	IgT	AACATCACCTGGCACATCAA	TTCAGGTTGCCCTTTGATTC	(20)
7	IgD	AGCTACATGGGAGTCAGTCAACT	CTTCGATCCTACCTCCAGTTCCT	(20)
8	IgM	CCTTAACCAGCCGAAAGGG	CCAACGCCATACAGCAGAG	(20)
9	IFNϒ	GAAGGCTCTGTCCGAGTTCA	TGTGTGATTTGAGCCTCTGG	(20)

The quantitative Real-Time PCR (qRT-PCR) was conducted using CFX96 Touch Real-Time PCR detection system (Bio-Rad, München, Germany). Trout beta-actin was used as a reference gene for normalization (23, 24).

The qRT-PCR assay was performed in a total volume of 10 μL containing 5 μL of 2× SsoAdvanced™ Universal SYBR Green SuperMix (Bio-Rad), 0.5 μL of forward and reverse primer, 3 μL of nuclease free water, and 1 μL of 1:5 dilution of cDNA samples for every gene for the Group UN and Group S. The PCR temperature cycling conditions for all investigated genes were as follows: initial pre-denaturation at 95°C for 15 min, followed by a denaturation at 94°C for 15 s; 50 cycles of denaturation at 55°C for 15 s, annealing at 72°C for 15 s, and elongation at 55°C for 31 s. The final cycle was followed by extension at 55°C for 5 s. Each qRT-PCR assay was performed in duplicate.

### 2.5. Statistics analysis

Data concerning plasma parameters were submitted to one-way analysis (ANOVA) using SPSS 25 (25) to show significant differences between Group S and Group UN. The means were compared using the Student–Newman–Keuls (SNK) test.

Concerning the qRT-PCR analysis, the expression of the genes of the Group UN and S was normalized to the geometric mean of the reference genes (ß-actin). The relative gene expression between the UN and S groups was calculated using the 2-DealtaDealta Ct method (mean expression level adjusted to 1). The statistical difference between groups was determined using the two-tailed unpaired Student’s *t*-test with Welch’s correction. Value of *p* < 0.05 was considered significant. Data were analyzed using R statistical software (version 3.5.1).

## 3. Results

### 3.1. Genes expression

All fish exhibited good body condition and skin integrity. The skin mucus appeared homogeneous, uniform and well-distributed in both Groups UN and S. The levels of expression of several immune genes related to T and B cells were tested and compared including those coding for CD8, IL-6, IL-10a, IL-8, CD4, IgT, IgD, IgM, and IFN-γ.

**Figure 1:**
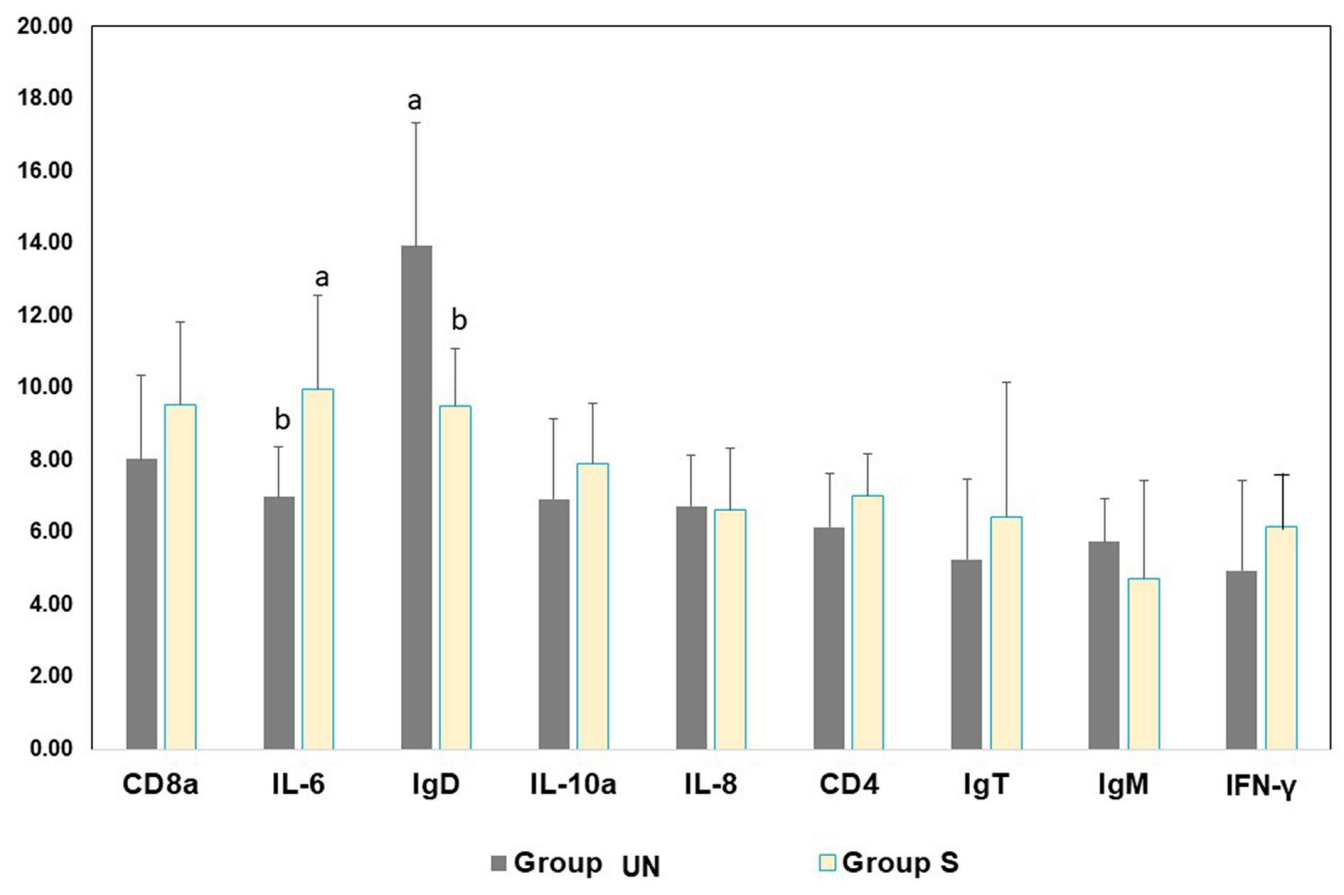
Gene expression level in skin mucus before (Group UN) and after (Group S) the slaughtering phase of the rainbow trout at the end of the productive cycle.

The expressions of the genes in rainbow trout are shown in Figure 1. A significant difference was found between the H and S rainbow trout groups with respect to IL-6 and IgD.

### 3.2. Plasma parameters

Table 2 shows the plasma metabolites measured in Group UN and Group S. Glucose values were significantly (*p* < 0.05) modulated. No significant differences were found comparing the average levels of cortisol and other metabolites between Group UN and Group S.

**Table 2:** Plasma parameters of rainbow trout before (Group UN) and after (Group S) the slaughtering phase (mean and standard deviation).

	Group UN	Group S
Cortisol (ng/mL)	12.67 ± 1.50	13.35 ± 1.11
Glucose (mg/dL)	81.56 ± 10.08 a	49.38 ± 7.52 b
Cholesterol (mg/dL)	266.82 ± 23.04	248.55 ± 9.39
Total triglycerides (mg/dL)	401.28 ± 102.54	511.52 ± 107.51
Total proteins (g/dL)	4.41 ± 0.61	4.18 ± 0.16
Albumin (g/dL)	1.79 ± 0.17	1.59 ± 0.15
Total globulins (g/dL)	2.62 ± 0.43	2.59 ± 0.11

Different letters (a, b) shows significant differences (*p* < 0.05).

## 4. Discussion

Previous studies emphasized the functions of skin mucus as a mechanical buffer and barrier against infections, while simultaneously fulfilling many other physiological functions (1, 7, 21). Other papers dealt with the effect of stress on gene expression and immune parameters in rainbow trout (26–28). The novelty of the current study is related to the effects of stress over on the skin mucus and blood metabolites of reared rainbow trout during the last phase of a standard farming cycle. However, the current study had some limitations represented by the low number of fish sampled and the two different cohorts of rainbow trout before (Group UN) and after (Group S) potentially stressful conditions. The logistic organization of samplings, performed during the steps of the productive cycle, made it difficult to sample. We sampled assisted by the operators of the trout company under COVID-19 restrictions while the production chain went on. Nevertheless, important findings emerged. The present trial indicated a low level of stress associated with this production system. An altered skin mucus activity and plasma glucose variation suggested that the management protocol was compatible with non-stressful farming conditions.

The gene expression of relevant stress- and immune-related transcripts indicated a significant difference between Group UN and Group S pertaining to IL-6 and IgD expression in fish skin mucus. IL-6 represents pro-inflammatory cytokines involved in the regulation of the immune system by controlling immunoglobulin production and the differentiation of lymphocytes and monocytes (29). IL-6 promotes the production of B lymphocytes (leukocytes responsible for the production of antibodies), and its participation in response to pathogens is well-known. Its effects were also tested in fish, including rainbow trout (21, 30). There are three classes of antibodies in fish: IgM, IgD, and IgT. The IgD class is an indicator of previous exposure to pathogens involved in the adaptive response by activating B cells, basophils, and mast cells for the production of active antimicrobial agents (23). Its prevalence in the Group S, along with the higher expression of IL-6 genes, suggests a reaction of the fish immune system to handling. However, considering that the differences between the blood cortisol levels of the UN and S groups were not significantly different, it can be inferred that pre-slaughter handling/processing did not induce stress in fish.

As concerns the plasma glucose, the Group S showed a depletion respect to the Group UN showing an average of around 39% below the basal values for this species (31). When fish are under stress conditions, alterations are expected with an increasing trend of its metabolism (32). However, sometimes a glucose decrease has been reported, as in the current study, at the end of the productive cycle. This reduction was considered related to the difficulty to maintain high glucose range due to the high request for glucose mobilization from the other tissues as observed in other studies (33, 34). This low value of glucose is compatible with the slower blood circulation that is documented when the blood sampling is performed after stunning (35). The absence of stress could be explained by the fact that animals, including fish can become habituated to repeated handling under farming conditions provided the intensity is mild and of short duration.

Rainbow trout are able to exhibit a cumulative response to certain repeated stressors in rearing (31). A modified and reduced physiological response can attenuate the involvement of the hypothalamus-pituitary-interrenal axis with a very limited plasma cortisol variation. Farmed fish can habituate to conditions associated with maintaining optimal water quality, cleaning of bottom race-ways, use of water pump system to sort fish and moderately high (20–35 kg/m^3^) stocking densities. These interactive factors are considered fundamental for fish welfare (36). Nevertheless, considering the experimental design and low sample size, further studies are needed with more specimens in order to show the degree of stress experienced by the fish in response to stressors can compromise welfare in farming conditions.

## Data availability statement

The datasets presented in this study can be found in online repositories. The names of the repository/repositories and accession number(s) can be found in the article/Supplementary material.

## Ethics statement

The animal studies were approved by Organismo Preposto al Benessere Animale University of Camerino. The studies were conducted in accordance with the local legislation and institutional requirements. Written informed consent was obtained from the owners for the participation of their animals in this study.

## Author contributions

EF, GM, and MS: conceptualization. EF and FA: methodology, software, and investigation. EF, MS, ME-M, and GM: formal analysis and project administration. EF, GM, FA, MS, and ME-M: resources. EF, AR, and MS: writing-original draft preparation and writing-review and editing. AR, MS, and ME-M: supervision. EF, ME-M, and AR: funding acquisition. All authors contributed to the article and approved the submitted version.

## Funding

This research was supported by Eureka Project 2019–2020. The funders had no role in the design of the study; in the collection, analyses, or interpretation of the data; in the writing of the manuscript; or in the decision to publish the results.

## Conflict of interest

The authors declare that the research was conducted in the absence of any commercial or financial relationships that could be construed as a potential conflict of interest.

## Publisher’s note

All claims expressed in this article are solely those of the authors and do not necessarily represent those of their affiliated organizations, or those of the publisher, the editors and the reviewers. Any product that may be evaluated in this article, or claim that may be made by its manufacturer, is not guaranteed or endorsed by the publisher.
